# Adolescent Trajectories of Aerobic Fitness and Adiposity as Markers of Cardiometabolic Risk in Adulthood

**DOI:** 10.1155/2017/6471938

**Published:** 2017-11-27

**Authors:** S. A. Jackowski, J. C. Eisenmann, L. B. Sherar, D. A. Bailey, A. D. G. Baxter-Jones

**Affiliations:** ^1^College of Kinesiology, University of Saskatchewan, Saskatoon, SK, Canada; ^2^Deparment of Radiology, College of Osteopathic Medicine, Michigan State University, East Lansing, MI, USA; ^3^School of Sport, Exercise and Health Sciences, Loughborough University, Loughborough, UK; ^4^School of Human Movement Studies, University of Queensland, Brisbane, QLD, Australia

## Abstract

**Purpose:**

The aim of this study was to investigate whether adolescent growth trajectories of aerobic fitness and adiposity were associated with mid-adulthood cardiometabolic risk (CMR).

**Methods:**

Participants were drawn from the Saskatchewan Growth and Development Study (1963–1973). Adolescent growth trajectories for maximal aerobic capacity (absolute VO_2_ (AbsVO_2_)), skinfolds (SF), representing total body (Sum6SF) and central adiposity (TrunkSF), and body mass index (BMI) were determined from 7 to 17 years of age. In mid-adulthood (40 to 50 years of age), 61 individuals (23 females) returned for follow-ups. A CMR score was calculated to group participants as displaying either high or a low CMR. Multilevel hierarchical models were constructed, comparing the adolescent growth trajectories of AbsVO_2,_ Sum6SF, TrunkSF, and BMI between CMR groupings.

**Results:**

There were no significant differences in the adolescent development of AbsVO_2,_ Sum6SF, TrunkSF, and BMI between adult CMR groupings (*p* > 0.05). Individuals with high CMR accrued 62% greater adjusted total body fat percentage from adolescence to adulthood (*p*=0.03).

**Conclusions:**

Growth trajectories of adolescent aerobic fitness and adiposity do not appear to be associated with mid-adulthood CMR. Individuals should be encouraged to participate in behaviours that promote healthy aerobic fitness and adiposity levels throughout life to reduce lifelong CMR.

## 1. Introduction

Cardiovascular disease, type 2 diabetes, and metabolic syndrome continue to be significant public health burdens in contemporary society [1]. Cardiometabolic risk (CMR) factors, such as elevated cholesterol, dyslipidemia, and insulin insensitivity, are well known to increase the risk of adult cardiometabolic diseases [2, 3]. These CMR factors have also been documented to track moderately well from childhood to adulthood [4–6], and their early life presence is a cause for concern as it may increase an individual's risk for cardiometabolic diseases [7–9].

Aerobic fitness [10–12] and adiposity [13–16], including obesity status [8, 17], are well documented to be independent predictors of mortality and CMR in adulthood. Maximal aerobic capacity or aerobic fitness is shown to be associated with improved cardiovascular function and reduced low-density lipoprotein cholesterol and total triglycerides (i.e., low CMR). Similarly, greater adiposity is positively associated with elevated total cholesterol, circulating triglycerides levels, insulin insensitivity, and overall CMR [7, 8]. Recent studies also suggest that the distribution of adipose tissue may play a critical role in the association of CMR factors, with adults carrying greater centrally located adipose tissue displaying elevated CMR profiles [14, 16]. In conjunction with adiposity, obesity status, often determined by body mass index (BMI), has received significant attention, as it is recognized to increase CMR, cardiometabolic diseases, and mortality [7, 8, 18, 19]. Despite these well-documented associations in adulthood, limited studies are available in youths and even fewer have prospective follow-ups that examine the longitudinal effects. The few longitudinal studies focusing on childhood obesity status have observed that higher BMI during childhood may predict long-term cardiometabolic disease in adulthood [7, 8, 19]; however, recent meta-analyses have questioned this conjecture concluding that there is little evidence to support the view that childhood obesity is an independent risk factor for adult CMR [20, 21]. Therefore, further prospective studies that assess not only obesity status but also aerobic fitness and adiposity from adolescence into adulthood are warranted to better understand the potential impact these risk factors have on adult CMR. There is increasing emphasis on the “lifecourse approach” to investigating adult chronic disease and health, and adolescence is considered a critical period when the timing, magnitude, and duration of growth greatly influence physical size, body composition, and physiological function [22]. It is also recognized that the antecedents of adult CMR may originate during this critical period [7, 10, 13, 14]. Yet, prospective longitudinal cohort studies that would firmly establish links between adolescent antecedents and adult health status are limited. A number of cross-sectional analyses have examined the association between adolescent aerobic fitness, obesity status, and cardiovascular risk factors in young adults, at 20–30 yrs of age, but the results are inconclusive [20, 21, 23–25]. This may be due to the limited length of follow-up into young adulthood (i.e., twenties) given that the clinical manifestations of cardiometabolic diseases often occur in mid-adulthood (i.e., 40–50 yrs) [26]. Furthermore, although previous studies have examined the cross-sectional associations [10, 13, 14, 17, 23–25], there remains a paucity of prospective data examining how the growth and development of these modifiable variables may influence subsequent adult health outcomes and CMR in mid-adulthood.

The Saskatchewan Growth and Development Study (SGDS) is one of the few projects which provide a unique opportunity to address the relationship between adolescent growth trajectories and subsequent adult health. This longitudinal growth study was initially carried out from 1964 to 1973 and included an evaluation of a number of annual physiological and growth parameters in children/adolescents. In two adult follow-up periods, participants' CMR was measured at ages 40 and/or 50 years. Thus, this longitudinal cohort is ideally suited to prospectively investigate the role that adolescent development of aerobic fitness, adiposity, and obesity status may have on CMR in mid-adulthood. The purpose of this study, therefore, was to determine if the developmental growth trajectories of aerobic fitness, adiposity, and obesity status were associated with CMR at 40–50 years of age in a cohort followed longitudinally for nearly four decades.

## 2. Materials and Methods

### 2.1. Participants

**Figure 1:**
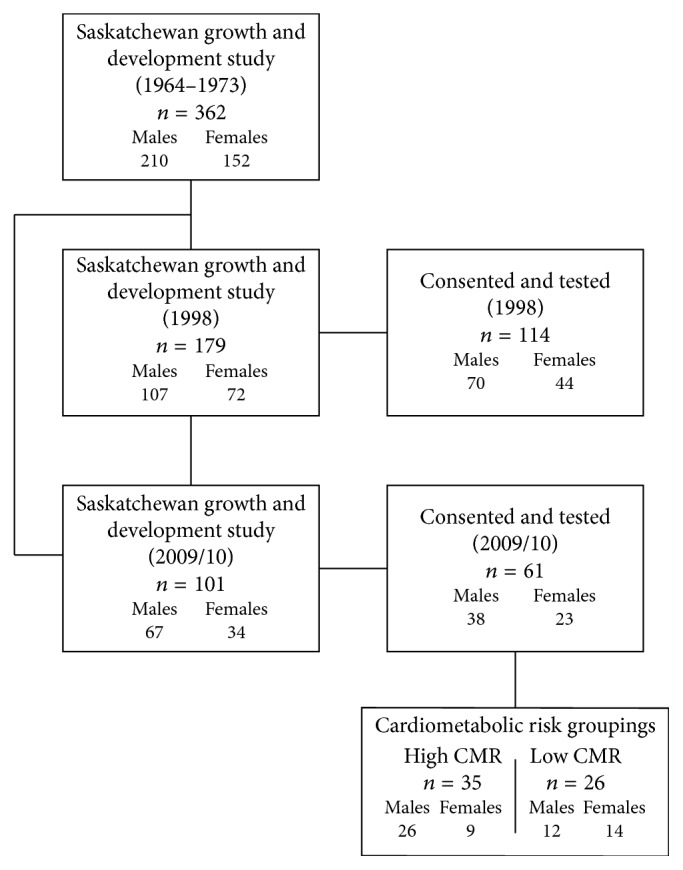
Participant numbers for the Saskatchewan Growth and Development Study and follow-ups in 1998 and 2009/10.

Participants were drawn from the Saskatchewan Growth and Development Study (SGDS, 1963–2010). The SGDS is a unique longitudinal growth study conceived in 1963, where youth were serially measured for up to ten years and then reassessed in 1998/99 and again in 2009/10. A full description of the original study can be found elsewhere [27, 28]. In brief, in 1964, the parents of a sample of 276 seven-year-old Caucasian males, randomly selected from the elementary school system from the City of Saskatoon, were contacted and invited to participate. Male participants were followed annually from 1964 to 1973, covering the age span of 7–17 years. In addition to the main group of males, in 1965 females were added in overlapping cohorts using a mixed longitudinal design and followed for 5 years. By 1973, 362 participants (214 males, 152 females) were tested on at least one occasion during childhood and/or adolescence (median 7 testing occasions). In 1998/99, male participants with > 9 years of adolescent data (*n* = 140) and females with > 3 years of adolescent data (*n* = 92) were invited to participate in a follow-up, when participants were approximately 40 years of age. One hundred seventy-nine participants (107 males, 72 females) were successfully located, of whom 70 males and 44 women consented and were tested [29]. In 2008, significant effort was made to recontact all participants. One hundred one (67 males, 34 females) were contacted and invited to participate. Sixty-one (38 males, 23 females) consented to take part and were tested between 2009 and 2010. A breakdown of participant's numbers can be found in Figure 1. All procedures and protocols were approved by the University of Saskatchewan's Biomedical Research Ethics Board, and written informed consent was given by each participant.

### 2.2. Anthropometry

Anthropometric measurements of height and weight were taken by trained personnel at each visit using the same protocols in both childhood/adolescence and adulthood. Height was measured to the nearest 0.1 cm on a wall-mounted stadiometer (Holtain Ltd., Crymych, Dyfed, UK), and weight was measured to 0.1 kg on a weight scale (Toledo Scale Company of Canada, Windsor, ON). In childhood/adolescence, each measurement was compared to the previous year and repeated if there appeared to be ambiguities. From 1998 onwards, two measurements were taken, with a potential third if more than a 0.4 cm/kg difference was observed between the first two measures. Body mass index (BMI) was calculated from height (ht) and weight (wt) (wt/ht^2^).

### 2.3. Skinfold Measurement

Adiposity was assessed from skinfold thickness measurements using a Harpenden skinfold caliper recorded to the nearest 0.1 mm by trained personnel following standardized procedures [27, 30, 31]. Skinfold measures were taken from 6 body regions: (1) suprailiac, (2) abdominal, (3) front thigh, (4) triceps, (5) subscapular, and (6) right chest (males)/rear thigh (females). These six measures were summed to generate an estimate of total body adiposity (Sum6SF). A trunk skinfold (TrunkSF) measure was ascertained from summing suprailiac and abdominal skinfold measures as a surrogate of central adiposity.

### 2.4. Aerobic Fitness Measures

During childhood and adolescence, aerobic fitness was measured by oxygen consumption (absolute VO_2_ (AbsVO_2_)) during a motorized treadmill protocol run to voluntary exhaustion [27, 28]. In brief, participants began by walking at a 0% grade at 4.8 km/hr for 3 minutes. Every 3 minutes subsequent, the treadmill speed was increased (9.6 km/hr, 14.4 km/hr, and finally 19.2 km/hr) until voluntary exhaustion. Expired air was collected, via a mouth piece, using a three-way rotating Douglas Bag valve. Respiratory gas samples were determined by oxygen and carbon dioxide analyzers. Ventilatory volumes were determined using a flowmeter. A full description of these testing methodology applied to the SGDS cohort can be found elsewhere [27, 28].

In adulthood, AbsVO_2_ was estimated from submaximal protocols. In 1998/99, the Bruce treadmill protocol was used. Participants were asked to walk/run on a motorized treadmill at increasing speeds and inclines every 3 minutes until voluntary exhaustion. Using the number of minutes that the participant was able to run, AbsVO_2_ was estimated using previously developed nomograms [32, 33]. In 2009/10, AbsVO_2_ was predicted using a submaximal cycle ergometer test following the Astrand protocol [34]. Participants cycled at a resistance determined by the standard Canadian Exercise Physiology Astrand protocols [34]. Using the participant's heart rate, weight, and resistance at which they pedaled, AbsVO_2_ was estimated. The estimate of AbsVO_2_ using a cycle ergometer test was due to safety concerns, and reflected participants at 50 years of age were not willing and/or capable of completing the previous maximal treadmill test.

### 2.5. Peak Height Velocity

To establish the age at peak linear growth (indexed as peak height velocity (PHV)) for each child, whole-year height velocity values were calculated for each participant using childhood and adolescent data by dividing the difference between the annual distance increments by the age increment. Preece-Baines Model 1 growth functions were applied to these data to ascertain the age of PHV [35]. Biological age for each participant was then calculated by subtracting the age of PHV from the decimal age at the time of measurement.

### 2.6. Smoking Habits, Nutritional Intake, and Physical Activity Assessments

In adulthood, participants were given health habits, food frequency, and subjective physical activity questionnaires to determine current medication use, smoking status, nutritional intake, and physical activity levels. Medication use was ascertained from a written questionnaire where participants were asked to record their current intake of prescription medications and over-the-counter supplements. Current smoking status was determined by a “YES/NO” question, with follow-up questions on previous smoking history, duration, and number of cigarettes consumed per day. Nutritional intake was ascertained by a semiquantitative questionnaire in which participants reported the frequency and portion size of ninety-nine food items. Adult physical activity was determined by the Physical Activity Questionnaire for Adults (PAQ-AD). The PAQ-AD is designed to assess the general physical activity levels in the previous seven days, scoring 7 items on a five-point Likert-type scale, where 1 is considered low activity and 5 is high levels of activity. The PAQ-AD had been previously reported to be a valid and reliable measure of physical activity levels in adults [36, 37].

### 2.7. Metabolic Assessments

In adulthood, a blood sample was drawn following a 12-hour overnight fast using standard venipuncture procedures by trained phlebotomists. Blood samples were centrifuged for 15 minutes at 1200*g*, alloquated into 0.5 ml microcentrifuge tubes, and stored at −70°C prior to analysis. Serum markers of total cholesterol, high-density lipoprotein, triglycerides, fasting glucose, and fasting insulin were measured using automated techniques by technicians at the Royal University Hospital (Saskatoon, SK, Canada).

### 2.8. Cardiometabolic Risk Score

A composite score was chosen to represent adult CMR because the number of participants classified with a cardiometabolic disease or metabolic syndrome was low. To account for variables known to influence metabolic status, serum metabolic markers (e.g. total cholesterol, high-density lipoprotein, triglycerides, fasting glucose, and fasting insulin) were regressed onto chronological age, sex, fiber intake, adult physical activity level, smoking status, and socioeconomic status, with the standardized residuals saved. The standardized residuals for total cholesterol, triglycerides, and glucose were summed to generate an adult cardiometabolic risk score (CMR score). Participants were then median split into a high or low CMR grouping from their derived CMR scores. If the participants had adult measurements from 1998/99 and 2009/10, then the data from 2009/10 were used to determine CMR score and grouping in the present analysis.

### 2.9. Statistical Analysis

To be included in the present analyses, participants required (i) a measure of age at peak height velocity (APHV), (ii) a measure of adolescence aerobic fitness, (iii) adolescence skinfold measures, and (iv) data to determine CMR in mid-adulthood (either 40 or 50 years of age). Sixty-four participants (52 males and 12 females) fulfilled these criteria and were retained for longitudinal analyses. Descriptive variables were determined for individuals during adolescence (at age of PHV) and in adulthood (either 40 or 50 years of age). A change variable was also calculated by subtracting the adolescence measures from the adult measures when available. Descriptive variables were assessed for normality using skewness and kurtosis, and any violations were adjusted using logarithmic transformations. Cross-sectional analyses to compare descriptive characteristics and change measures between adult CMR groups were assessed using independent *t*-test, for normalized data, and Mann–Whitney *U* test, when normality violations could not be adjusted. Cross-sectional analyses were performed using SPSS version 23.0 (IBM, Statistical Package for Social Sciences, Chicago, IL). All values are presented as means ± standard deviation unless otherwise specified.

For the longitudinal analyses, multilevel (hierarchical) random effects models were constructed using a multilevel modelling approach (MlwiN version 2.30, Multilevel Models Project; Institute of Education, University of London, UK). Detailed description of multilevel modelling as applied to other longitudinal growth datasets has been previously reported [38, 39]. For the present analyses, growth trajectories of aerobic fitness (AbVO_2_) and adiposity (Sum6SF, TrunkSF, and BMI) were calculated repeatedly within (level 1 of the hierarchy) and between individuals (level 2 of the hierarchy). Models were built in a stepwise procedure, with predictor variables, biological age, height, and sex added one at a time, to predict aerobic AbVO_2_, Sum6SF, TrunkSF, and BMI development and test if difference existed between CMR groups. A total of 4 independent multilevel random effects models were constructed for aerobic fitness, adiposity, and obesity parameters (AbVO_2_, Sum6SF, TrunkSF, and BMI) with biological age, biological age^2^, height, sex, and CMR grouping included as predictor variables. Further details of the multilevel random effects modelling used in the present study are found in Supplementary Material available online at https://doi.org/10.1155/2017/6471938.

## 3. Results

Descriptive characteristics in adolescence and adulthood and changes from adolescence to adulthood for individuals categorized as having low and/or high CMR are displayed in Table 1. As expected, high CMR participants had significantly higher adult serum levels of fasting glucose, total cholesterol, total triglycerides, and low-density lipoprotein than their low CMR peers (*p* < 0.05). Additionally, the high CMR participants had significantly greater changes in their total body fat percentage from adolescence to adulthood (*p* < 0.05). No significant differences were observed for any other variables at the age of PHV or in adulthood between CMR groupings.

### 3.1. Longitudinal Analyses

Table 2 summarizes the results from the multilevel model analyses for the development of AbVO_2_, Sum6SF, TrunkSF, and BMI between low and high CMR categorized individuals. For all models, the significant random effects variances at level 1 of the models indicate that AbVO_2_, Sum6SF, TrunkSF, and/or BMI were increasing significantly at each measurement occasion within individuals (Constant, *ε*_*ij*_ > 2∗SEE; *p* < 0.05). The between-individuals random effects variance matrix (level 2) for each model indicates that individuals had significantly different AbVO_2_, Sum6SF, TrunkSF, and/or BMI growth curves, both in terms of their intercepts (constant, *µ*_*j*_, *p* < 0.05), and the slopes of their lines (biological age, *ν*_*j*_*X*_*ij*_, *p* < 0.05). To shape the individuals' curves, power functions of biological age (biological age^2^) were added as fixed effects, to allow for the nonlinearity of growth. Although these power functions were not significant in all models, they were retained in all models as they improved the model fit as indicated by log-likelihood ratio statistics.

**Figure 2:**
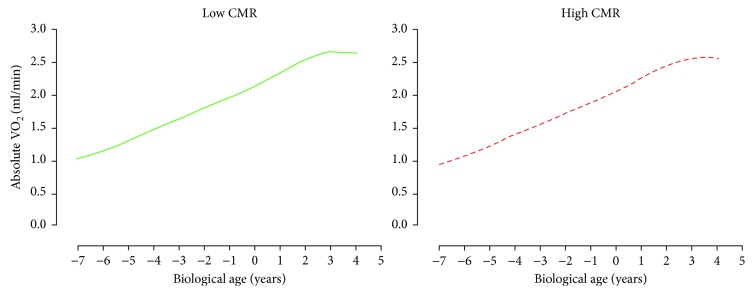
Predicted adolescent absolute VO_2_ growth trajectories for individuals categorized as having low and high cardiometabolic risk (CMR) in adulthood. Predicted models adjusted for biological age, height, and sex.

**Figure 3:**
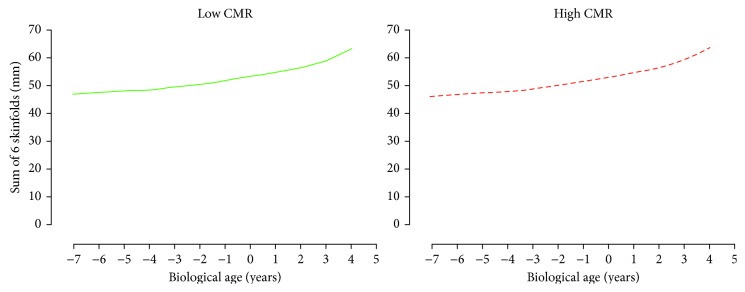
Predicted adolescent sum of six skinfolds growth trajectories for individuals categorized as having low and high cardiometabolic risk (CMR) in adulthood. Predicted models adjusted for biological age, height, and sex.

**Figure 4:**
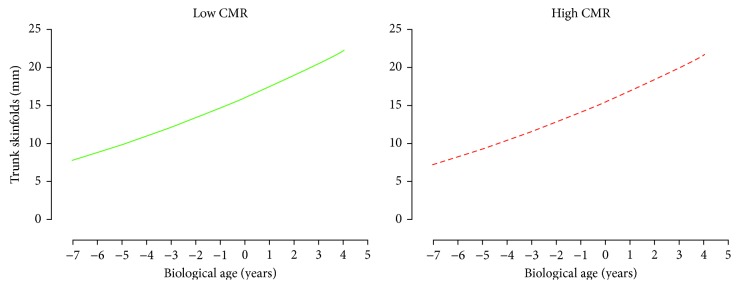
Predicted adolescent trunk skinfolds growth trajectories for individuals categorized as having low and high cardiometabolic risk (CMR) in adulthood. Predicted models adjusted for biological age, height, and sex.

**Figure 5:**
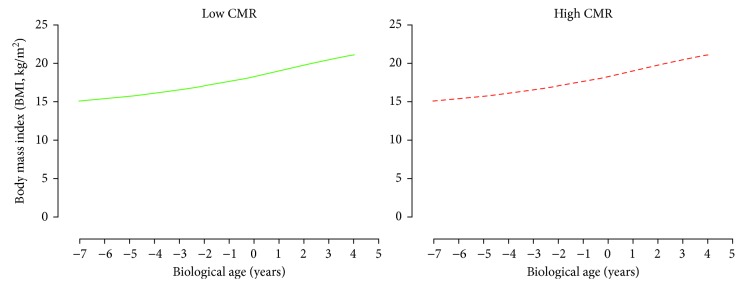
Predicted adolescent body mass index growth trajectories for individuals categorized as having low and high cardiometabolic risk (CMR) in adulthood. Predicted models adjusted for biological age, height, and sex.

For AbVO_2_, multilevel model analyses revealed that only height was the significant predictor of AbVO_2_ development (*p* < 0.05). There was no significant difference in the development of AbVO_2_ between the adult CMR groupings after accounting for biological age, height, and sex (*p* < 0.05, Figure 2). For both skinfold measurements (Sum6SF and TrunkSF), only biological age was a significant predictor (*p* < 0.05). There were no significant differences in the development of Sum6SF and TrunkSF between adult CMR groupings (*p* < 0.05, Figures 3 and 4). Biological age and height were observed to be significant predictors in the development of adolescent BMI (*p* < 0.05); however, there were no significant differences in BMI development between CMR groupings once adjusting for biological age, height, and sex (*p* < 0.05, Figure 5).

## 4. Discussion

The aim of this study was to investigate whether adolescent growth trajectories of aerobic fitness and adiposity were associated with adult CMR at 50 years of age. It was observed that the longitudinal development of aerobic fitness, skinfold thickness, and BMI did not differ between individuals classified as displaying significantly different CMR profiles in adulthood. However, those who accrued greater adjusted body fat percentage from adolescent to adulthood displayed greater adult CMR. Although several studies have speculated about the relationships between adolescent fitness and fatness, and adult CMR, this is the first study, to our knowledge, to prospectively investigate whether adolescent growth trajectories of aerobic fitness and adiposity differed between individuals with varying adult cardiometabolic risk profiles, using a longitudinally followed cohort for nearly 40 years.

### 4.1. Adolescent Aerobic Fitness

Aerobic fitness has been documented to impact a host of markers of cardiovascular and metabolic diseases in both adolescents and adults [10–12]. Few studies, however, exist that prospectively examine the longitudinal role of adolescent physical fitness on adult CMR [17, 23–25], and none, that the authors are aware of, span into mid-adulthood when the manifestations of cardiometabolic diseases become evident. Of the limited longitudinal observations, the impact of adolescent aerobic fitness on adult CMR markers is equivocal. Twisk et al. [25] and Boreham et al. [23] both describe modest positive associations between adolescent aerobic fitness and serum lipid levels in early adulthood, while Hasselstrom et al. suggest that adolescent aerobic fitness is a poor predictor of adult CMR but emphasizes that the change in aerobic fitness from adolescence to adulthood plays a strong negative impact on early adulthood cardiovascular risk [24]. In the present study, both the longitudinal associations of aerobic fitness development and the change in aerobic fitness were examined. It was observed that the neither were associated with CMR in mid-adulthood. These observations conflict previous studies and may be a result of the present study's lengthier observation period. We have shown in this cohort that tracking of aerobic fitness diminishes over time, with poor associations documented between adolescent and adult aerobic fitness in mid-adulthood [40], and it is this poor aerobic fitness tracking that may also be limiting any association between adolescent aerobic fitness development and adult cardiometabolic profiles. Additionally, 75% of the returning SGDS participants displayed estimated relative VO_2_ max values below 35 ml/kg/min in adulthood (data not shown), highlighting the poor estimated aerobic capacity of these individuals at nearly 40/50 years of age. Thus, any adolescent associations in adult aerobic fitness may be weakened as the measurement interval increased, suggesting adult CMR may be more reflective of the participant's current diminished aerobic fitness rather than the development of their adolescent aerobic fitness. This conjecture is consistent with prior studies which have observed that low aerobic fitness in adulthood was strongly associated with short-term CMR mortality and that the strength of these associations significantly weakened after three decades of follow-up [41]. The American Heart Association and American College of Cardiology have also stated that the evidence supporting the contribution of cardiorespiratory fitness to risk models of cardiovascular health is inconclusive, promoting instead general physical activity as a more important health behavior for lifelong CMR [42]. Though the present study cannot address the latter, it does support questioning the predictive value of adolescent aerobic fitness on adult CMR. Future research capable of parsing aerobic fitness and physical activity levels is necessary to identify any potential unique contributions of both variables on adult CMR.

### 4.2. Adolescent Adiposity

In addition to aerobic fitness, adiposity, during both adolescent and adulthood, has been widely documented to impact CVD, CMR, and overall mortality [8, 11, 13–16, 20]; however, few prospective longitudinal studies exist spanning from childhood to mid-adulthood. In the present study, two approaches were employed to investigate the relationship between adolescent adiposity development and adult CMR. The first was to examine the distribution of adipose tissue, as it has been suggested that individuals carrying greater centrally located adipose tissue display elevated adult CMR [14, 16]. The trajectories of trunk adiposity and the sum of six skinfolds were ascertained as representations of central and total body adiposity, respectively. Neither expression of adiposity was observed to be associated with adult CMR profiles. These findings are in opposition to Sherar and colleagues who observed that when young adults (20–30 years of age) were similarly dichotomized as having low or high CMR, adolescent trunk adiposity accrual was significantly greater in those individuals displaying high adult CMR profiles [14]. The discrepancies between findings may be attributed to differences in determining central adiposity, as Sherar and coauthors employed dual energy X-ray absorptiometry (DXA) versus skinfold assessments. DXA measurements can provide a better estimate of both visceral and subcutaneous adipose tissue, while skinfold measurements only provide an estimation of subcutaneous adiposity. Thus, the present study observations suggest that the development of adolescent subcutaneous adipose tissue, whether at the trunk or whole body, may not be an imperative indicator of adult CMR. Instead, centrally located visceral adiposity may be the more important indicator of future CMR. Additionally, there are generational differences between birth cohorts that may play an important role in the discrepancies observed. The SGDS cohort has significantly lower fat mass profiles compared to more contemporary and regionally similar birth cohorts [43] and is in agreement with secular trend data from Canada that has shown a significant increase in weight, BMI, and waist circumference [44–46]. Given these variables are documented to be associated with CMR, newer generations may be predisposed to lengthier exposure to risk factors for cardiometabolic diseases not observed in earlier generational cohorts. These suppositions are difficult to substantiate and can only be supported by the continued investment in prospective longitudinal cohorts for comparison.

Similar to models of adipose distribution, adolescent BMI growth trajectories were not found to differ between individuals with low and high adult CMR. These observations also contradict previous adolescence and adulthood findings which have reported significant associations with increased childhood and adolescence BMI on cardiovascular risk profiles [9]. These differences, however, may be a result of the period of BMI development investigated. Boyer and colleagues reported that early life BMI development was associated with adolescent CVD, emphasizing that the period around the adiposity rebound (5-6 years of age) is vital to adolescent adipose tissue development and future cardiovascular risk [9]. Similarly, studies have also documented the positive association with an early adiposity rebound and increased cardiovascular risk factors in adulthood [47, 48]. This might suggest that the present study did not capture the crucial period of BMI development and that earlier childhood investigations may be fundamental in establishing links between BMI development and lifelong CMR. Despite these observations in early childhood, recent systematic reviews have questioned adolescent associations between BMI and adult CVD outcomes [20, 21] and are in agreement with the present study's findings. These reviews concluded that contemporaneous adult markers of BMI are the better predictor of current markers of triglycerides, insulin insensitivity, and metabolic syndrome [20, 21]. Although it has been documented that adolescent BMI tracks into adulthood, especially amongst individuals who have high BMI values [49], it should be noted that the SGDS cohort (circa 1970, preobesity epidemic) was a relatively healthy population with few individuals displaying adolescent overweight or obese BMI profiles. Thus, their “healthy” BMI status during adolescence may have reduced their exposure to this key CMR marker and diminished observable associations with adult CMR. Additionally, the SGDS's low adolescent overweight and obesity prevalence arguably limit the generalization of the present findings to more modern cohorts, where obesity and reduced physical activity levels are more widely prevalent. These modern cohorts may therefore be exposed to lengthier periods of elevate risk factors, which in turn, may lead to increased CMR [7, 8]. Yet, despite the SGDS's low adolescent obesity prevalence, many participants displayed high adult CMR, showcasing that a “healthy” adolescent BMI profile may not be protective from developing elevated CMR in adulthood. Two-thirds of the individuals who participated in the follow-up studies were classified as being overweight or obese as adults (data not shown), providing further support that current BMI status may be more representative of contemporaneous CMR than previous adolescent profiles.

### 4.3. Limitations

Though this is the first prospective longitudinal study to investigate whether adolescent growth trajectories of aerobic fitness and adiposity were associated with adult CMR at 50 years of age, the present study is not without limitation. The small sample size of the returning cohort at 50 years of age may (1) predispose the current conclusions to selection bias, (2) challenge the representative nature of this cohort, and (3) restrict the evaluation of individuals with contrasting cardiometabolic profiles. In addressing the first two items, it should be noted that a significant effort was made to contact and invite all original SGDS participants for the follow-up assessments in the present study, yet less than 20% of all original participant were included in the present analysis. Thus, selection bias remains an unavoidable limitation and may limit conclusions drawn. Attrition analyses were conducted to compare the adolescent characteristics and growth trajectories of individuals who returned to participate in the present follow-up study against their nonreturning peers (Supplementary Tables 1 and 2). It was observed that there were no significant differences in any adolescent characteristics or growth trajectories, other than individuals who returned being significantly taller, between these grouping of individuals. This appears to suggest that though the present study may be limited by a small sample size, these individuals are representative of the adolescent cohort from which they were derived. Finally, cardiometabolic risk profiles vary considerably between adults and between sexes. To account for this variation, a continuous cardiometabolic risk index was generated and used to categorize individuals into risk groupings. Though these adult groupings are observed to display significantly different cardiometabolic risk profiles, they may still be too homogeneous to truly observe potential sex differences resulting from adolescent aerobic fitness and adiposity development. Continued prospective longitudinal investigations are needed, and ones using a more comprehensive list of cardiometabolic risk factors, to further examine the early antecedents of aerobic fitness and adiposity on long-term health.

### 4.4. Conclusions

In conclusion, this longitudinal analysis showed that the adolescent development of absolute VO_2_ max, total body and centrally located subcutaneous adiposity, and BMI were not associated with adult CMR profiles at nearly 50 years of age. The relatively aerobically fit and healthy weight status of the present cohort may have resulted in reduced exposure to key CMR factors during adolescence and may limit its representation to more contemporary cohorts. Individuals should be encouraged to continue to participate in behaviours that promote and maintain healthy aerobic fitness and adiposity levels throughout life in order to reduce CMR.

## Supplementary Material

Statistical method for longitudinal analysis

## Figures and Tables

**Table 1:** Descriptive variables for participants categorized as having low and high adult cardiometabolic risk. Presented as mean ± standard deviation.

	Low CMR *n* = 26	High CMR *n* = 35	Difference (high versus low)	*p* value
CMR score	−1.67 ± 0.94	1.64 ± 0.91	3.31	**>0.01**
Fast glucose (mmol/l)	5.02 ± 0.38	5.57 ± 0.56	0.55	**>0.01**
Total cholesterol (mmol/l)	4.82 ± 0.76	6.10 ± 0.97	1.27	**>0.01**
Total triglycerides (mmol/l)	1.05 ± 0.45	2.02 ± 1.17	0.97	**>0.01**
HDL cholesterol (mmol/l)	1.39 ± 0.46	1.22 ± 0.39	−0.17	0.11
LDL cholesterol (mmol/l)	2.95 ± 0.63	3.97 ± 0.73	1.02	**>0.01**
PA score (1 low, 5 high)	1.98 ± 0.72	2.15 ± 0.69	0.17	0.34
SES category (1 low, 9 high)	6.69 ± 1.44	6.40 ± 1.63	−0.29	0.46
Fiber index	8.07 ± 2.19	7.99 ± 3.12	−0.07	0.92
Adult age (yrs)	48.62 ± 5.24	45.99 ± 6.35	−2.63	0.08
Age of PHV (yrs)	13.80 ± 1.44	13.16 ± 1.79	−0.64	0.13
Height at PHV (cm)	161.74 ± 9.07	158.72 ± 10.26	−3.02	0.22
Adult height (cm)	178.63 ± 8.48	174.57 ± 7.98	−4.06	0.05
Height change (cm)	16.89 ± 4.02	15.85 ± 5.13	−1.04	0.38
Weight at PHV (kg)	48.37 ± 9.20	45.78 ± 8.81	−2.60	0.25
Adult weight (kg)	86.63 ± 21.58	83.63 ± 15.96	−3.00	0.53
Weight change (kg)	38.26 ± 15.37	37.85 ± 11.25	−0.40	0.90
BMI at PHV (kg/m^2^)	18.34 ± 2.31	18.03 ± 1.97	−0.32	0.56
Adult BMI (kg/m^2^)	26.89 ± 5.10	27.29 ± 4.01	0.41	0.72
BMI change (kg/m^2^)	8.54 ± 4.10	9.27 ± 3.09	0.72	0.43
Absolute VO_2_ at PHV (ml/min)	2.65 ± 0.54	2.45 ± 0.53	−0.21	0.15
Adult absolute VO_2_ (ml/min)	3.10 ± 1.05	2.96 ± 0.94	−0.14	0.59
Absolute VO_2_ change (ml/min)	0.37 ± 0.86	0.53 ± 0.96	0.15	0.54
Relative VO_2_ at PHV (ml/kg/min)	54.69 ± 6.36	52.55 ± 7.34	−2.15	0.25
Adult relative VO_2_ (ml/kg/min)	29.86 ± 9.35	30.12 ± 8.78	0.26	0.91
Relative VO_2_ change (ml/kg/min)	−26.22 ± 7.89	−23.38 ± 9.59	2.85	0.25
LBM at PHV (kg)	40.57 ± 5.20	40.66 ± 5.84	0.10	0.95
Adult LBM (kg)	62.22 ± 12.34	59.92 ± 10.92	−2.30	0.44
LBM change (kg)	24.26 ± 7.98	23.72 ± 6.90	−0.54	0.80
Percent body fat at PHV (%)	18.00 ± 4.81	17.07 ± 3.59	−0.93	0.44
Adult percent body fat (%)	24.66 ± 10.02	27.82 ± 7.50	3.16	0.16
Percent body fat change (%)	5.80 ± 9.72	9.45 ± 5.11	3.65	**>0.05**
Sum6SF at PHV (mm)	55.92 ± 24.88	52.51 ± 16.66	−3.41	0.52
Adult sum6SF (mm)	131.16 ± 59.04	143.34 ± 51.19	12.18	0.40
Sum6SF change (mm)	73.78 ± 57.66	90.83 ± 49.02	17.05	0.22
TrunkSF at PHV (mm)	16.96 ± 8.31	15.09 ± 5.50	−1.87	0.29
Adult TrunkSF (mm)	98.80 ± 52.93	104.06 ± 37.15	5.26	0.65
TrunkSF change (mm)	81.43 ± 50.84	88.97 ± 35.22	7.54	0.17

CMR = cardiometabolic risk; HDL = high-density lipoprotein cholesterol; LDL = low-density lipoprotein cholesterol; PA score = physical activity score; SES; socioeconomic status; PHV = peak height velocity; BMI = body mass index; LBM = lean body mass; SF =  skinfolds.

**Table 2:** Multilevel regression models for aerobic fitness, adiposity, and obesity status measures.

Variable	AbsVO_2_	Sum6SF	TrunkSF	BMI
*Fixed effects*				
Constant	−2.52 ± 1.02	97.81 ± 24.66	17.28 ± 7.98	12.85 ± 1.94
Biological age (yrs)	NS	3.25 ± 1.03	1.45 ± 0.35	0.45 ± 0.08
Biological age^2^ (yrs)	NS	−0.13 ± 0.05	NS	NS
Height (cm)	0.03 ± 0.01	NS	NS	0.35 ± 0.01
Sex	NS	NS	NS	NS
CMR group	NS	NS	NS	NS
*Random effects*				
Level 1				
Constant (*ε*_*ij*_)	0.20 ± 0.02	82.01 ± 5.40	13.29 ± 0.87	0.38 ± 0.03
Level 2				
Constant (*µ*_*j*_)	0.31 ± 0.06	333.32 ± 61.93	42.71 ± 8.03	3.84 ± 0.69
Biological age (*ν*_*j*_*X*_*ij*_)	0.04 ± 0.01	35.99 ± 8.12	5.71 ± 1.17	0.31 ± 0.07
Constant∗biological age (*µ*_*j*_∗*ν*_*j*_*X*_*ij*_)	0.009 ± 0.002	6.36 ± 1.36	0.89 ± 0.19	0.04 ± 0.01

All numerical values are reported as significant, *p* < 0.05 (mean > 2∗SEE). NS = not significant and variable removed from the final model. Fixed effect values are estimated mean coefficients ± SEE (standard error estimate) for absolute VO_2_ (AbsVO_2_, ml/min), sum of six skinfolds (Sum6SF, mm), trunk skinfolds (TruckSF, mm), and body mass index (BMI, kg/m^2^). Random effects values are estimated mean variance ± SEE. Biological age is years centered around peak height velocity years of age (yrs). Height (cm); sex (male = 0, females = 1). Cardiometabolic risk group (CMR group; 0 = low CMR, 1 = high CMR).
