# Supplementary material for: Adolescent Trajectories of Aerobic Fitness and Adiposity as Markers of Cardiometabolic Risk in Adulthood
Source: J Obes. 2017 Nov 27;2017:6471938. doi: 10.1155/2017/6471938 (PMC5723934; doi:10.1155/2017/6471938)
Supplement: Supplementary file 1 — Statistical method for longitudinal analysis [file 6471938.f1.docx]

**Supplementary Materials**

Statistical Methods for Longitudinal Analysis

For the longitudinal analyses, multilevel (hierarchical) random effects models were constructed using a multilevel modelling approach (MlwiN version 2.30, Multilevel Models Project; Institute of Education, University of London, UK). Detailed description of multilevel modelling as applied to other longitudinal growth datasets has been previously reported (38) and complete details of this approach are described elsewhere (39). For the present analyses, growth trajectories of aerobic fitness (AbVO_2_) and adiposity (Sum6SF, TrunkSF, and BMI) were calculated repeatedly within (level 1 of the hierarchy) and between individuals (level 2 of the hierarchy). Analysis models that contain variables measured at different levels of a hierarchy are known as multilevel regression models. Specifically, the following additive random effects multilevel regression models were adopted to describe the developmental changes in aerobic fitness and adiposity parameters with age.

Equation 1. y_ij_ = (α+µ_j_)+ (β+ν_j_ ) x_ij_ + (z_1_ij_ + z_2_ij_ +…+ z_n_ij_) + ε_ij_

which can be reorganized to

Equation 2. y_ij_ = (α+β_j_x_ij_) + ( z_1_ij_ + z_2_ij_ + …+ z_n_ij_) + (µ_j_+ ν_j_ x_ij_ + ε_ij_).

where: y is the measurement parameter (AbVO_2_, Sum6SF, TrunkSF, and BMI) on measurement occasion i in the j-th individual; α is a constant; β_j_x_ij_ is the slope of the measurement parameter (AbVO_2_, Sum6SF, TrunkSF, and BMI) over time (in this model age is centered around the age of PHV or biological age zero) for the j-th individual; and z_1 to z_n are the coefficients of various explanatory variables (i.e. biological age, height, sex, and CMR grouping) at assessment occasion i in the j-the individual. These are the fixed parameters in the model.

µ_j_ , ν_j_x_ij_ and ε_ij_ are random quantities, whose means are equal to zero; they form the random parameters in the model. They are assumed to be uncorrelated and follow a normal distribution and thus their variances can be estimated; ε_ij_~N[0,var(ε_ij_)] is the level-1 residual (within individual) variance for the i-th assessment of the measurement parameter in the j-th individual; µ_j_~N[0,var(μ)] is the between individual intercept variance and v_j_x_ij_~N[0,var(v^j^x_ij_)] the between individuals slope variance; these are the level-2 residuals (between subjects) variances for the j-th individual. µ_j_*v_j_x_ij_~N[0,var(µ_j_*v_j_x_ij_)] explains the intercept-slope covariance relationship amongst the intercepts and slopes in the model.

Models were built in a stepwise procedure, i.e. predictor variables (z -fixed effects) were added one at a time, and the log likelihood ratio statistics was used to judge the fit of the model. Biological age (βjxij - centered around PHV, biological age zero) was added as both a random (level 1) and a fixed variable. This permits individuals to have independent intercepts and slopes and a calculation of the intercept-slope covariance relationship. A significant biological age coefficients (β) at level 1 of the models indicates that the aerobic fitness, adiposity, and/or obesity measure is increasing significantly at each measurement occasion within individuals. Significant coefficients at the individuals variance matrix (level 2 ) in each model indicates that individuals have significantly different growth curves for aerobic fitness, adiposity, and/or obesity measures, both in terms of their intercepts and the slopes and that the there is a relationship between intercepts and slopes in the model. Predictor variables (z) were accepted as significant if the estimated mean coefficient was greater than twice the standard error of the estimate (SEE, i.e. p < 0.05). If the retention criteria were not met the predictor variable was discarded. The power function biological age centered^2^ was introduced into the linear models to allow for the non-linearity of growth and was retained whether or not it was significant so as to shape the developmental curves. The predictor variable coefficients biological age, height, sex were used to predict aerobic AbVO_2_, Sum6SF, TrunkSF, and BMI development and test if difference existed between CMR groups with. A total of 4 independent multilevel (hierarchical) random effects models were constructed for aerobic fitness, adiposity, and obesity parameters (AbVO_2_, Sum6SF, TrunkSF, and BMI) with biological age, biological age^2^, height, sex and CMR grouping included as the predictor variables.
