# Supplementary material for: Adolescent Trajectories of Aerobic Fitness and Adiposity as Markers of Cardiometabolic Risk in Adulthood
Source: J Obes. 2017 Nov 27;2017:6471938. doi: 10.1155/2017/6471938 (PMC5723934; doi:10.1155/2017/6471938)
Supplement: Supplementary file 2 [file 6471938.f2.pdf]

Supplementary figures

Mouse Wellbeing and General Condition Score

			Mouse Number																															
		Observation	1st	2nd	3rd	4th	1st	2nd	3rd	4th	1st	2nd	3rd	4th	1st	2nd	3rd	4th	1st	2nd	3rd	4th	1st	2nd	3rd	4th	1st	2nd	3rd	4th	1st	2nd	3rd	4th
Fur	0	not alt.																																
	1	blunt/dirty																																
Posture	0	not alt.																																
	1	hunched																																
	2	flat and/or tremors																																
Mobility	0	not alt.																																
	1	slow/stiff movement when disturbed																																
	2	no movement when disturbed																																
Alertness	0	not alt.																																
	1	decreased																																
	2	no reaction																																
Weight	0	loss of 0.6g or more																																
	1	no loss to 0.5g loss																																
	2	any gain																																
Startle reflex	0	not alt.																																
	1	delayed																																
	E	none																																
		Total score																																

8 or more points = euthanasia

not alt. = not altered

time points of observation:

1st

2nd

3rd

time points of observation:

4th

5th

6th

Supplementary Fig. 1. Score sheet for a custom-developed mouse clinical assessment scoring system. The well-being of mice is assessed based on six endpoints every 12 hours (beginning at 12h post-CLP); tree severity grades (i.e. 0, 1 or 2 points) are assigned. Euthanasia is indicated when: score ≥8 and/or by inability to trigger the startle reflex and/or BT<28°C (recorded in at least two sequential measurements).

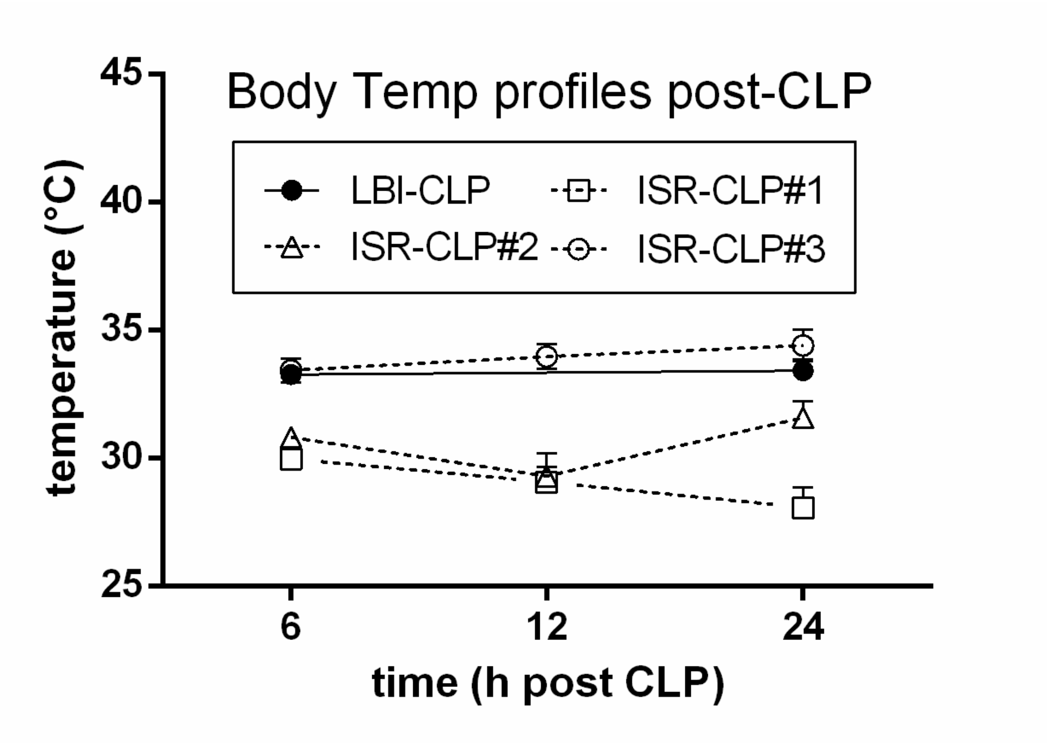

Supplementary Fig. 2. Comparison of body temperature (BT) profiles in CLP experiments performed in two different laboratories. BT profile was used as a surrogate of outcome to optimally standardize the magnitude of CLP severity between the surgery performed in Ludwig Boltzmann Institute (LBI; Vienna, Austria) and Institute of Surgical Research (ISR), University of Szeged (Hungary). Mice underwent three CLP runs at ISR and their BT profiles (at 6h, 12h and 24h) were compared to the BT profile (at 6 and 24h post-CLP) of the CLP mice enrolled in the survival study performed in LBI (solid line/dot). ISR-CLP #3 was designated as the best match and expanded to n=28 for further mitophagy analysis. LBI-CLP n=90-78; ISR-CLP#1 n=28-16; ISR-CLP#2 n=25-13; ISR-CLP#3 n=27-26. Data points shown as mean±SEM.

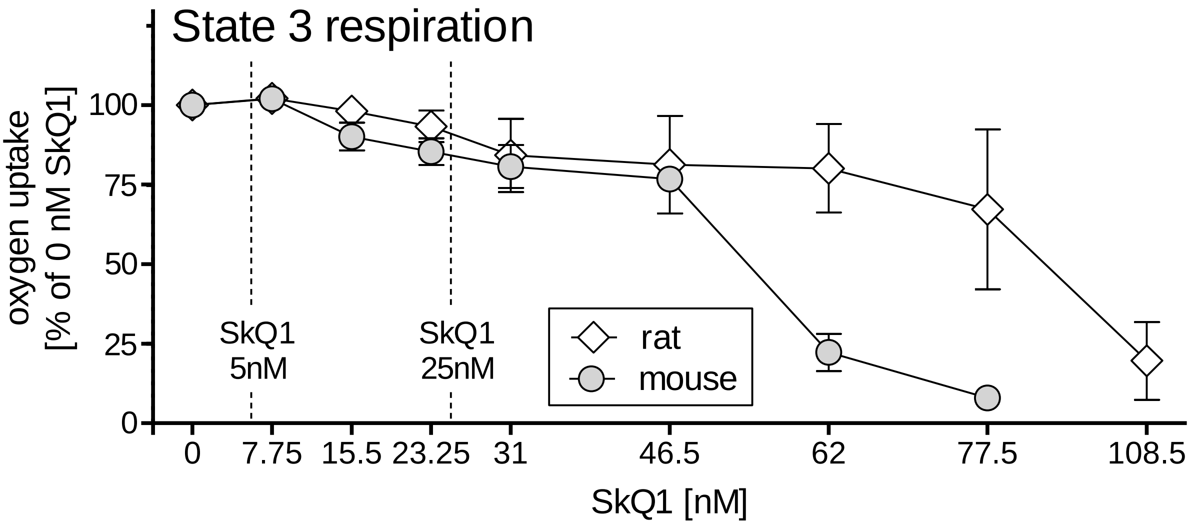

Supplementary Fig. 3. Effect of SkQ1 treatment on the state 3 mitochondrial respiration in the rat and mouse liver homogenates. State 3 respiration was measured in mouse and rat liver homogenates subjected to SkQ1 in the range of concentrations from 0 to 108.5nM. n=4/each species. Data points shown as mean±SD. Dotted lines indicate either the single (5nM) or cumulative (25nM) SkQ1 dose administered to CLP mice in the main survival study (Experiment 1). The rat data serve as species comparison.

Materials and Methods

Measurement of mitochondrial respiration in liver homogenate

Fifty milligrams of rat/mouse liver were homogenized (RW 1 basic homogenizer, IKA, Wilmington, NC, USA) with ten volumes (1:11 wt/vol) of buffer (0.25M saccharose, 10mM Tris, 0.5 mM EDTA, 5 mg/ml fatty acid-free bovine serum albumin, pH 7.2). For measurement of mitochondrial respiration, 25µL of liver homogenate was incubated in buffer containing 105mM KCl, 5mM KH_2PO_4, 20mM Tris-HCl, 0.5mM EDTA and 5 mg/ml fatty acid-free bovine serum albumin (pH 7.2, 37°C). State 3 respiration was monitored using a high-resolution respirometer (Oxygraph-2k, Oroboros Instruments, Austria) by addition of 10mM succinate, 1 ng/mL rotenone and 1mM ADP. All reagents were obtained from Sigma-Aldrich (Vienna, Austria).

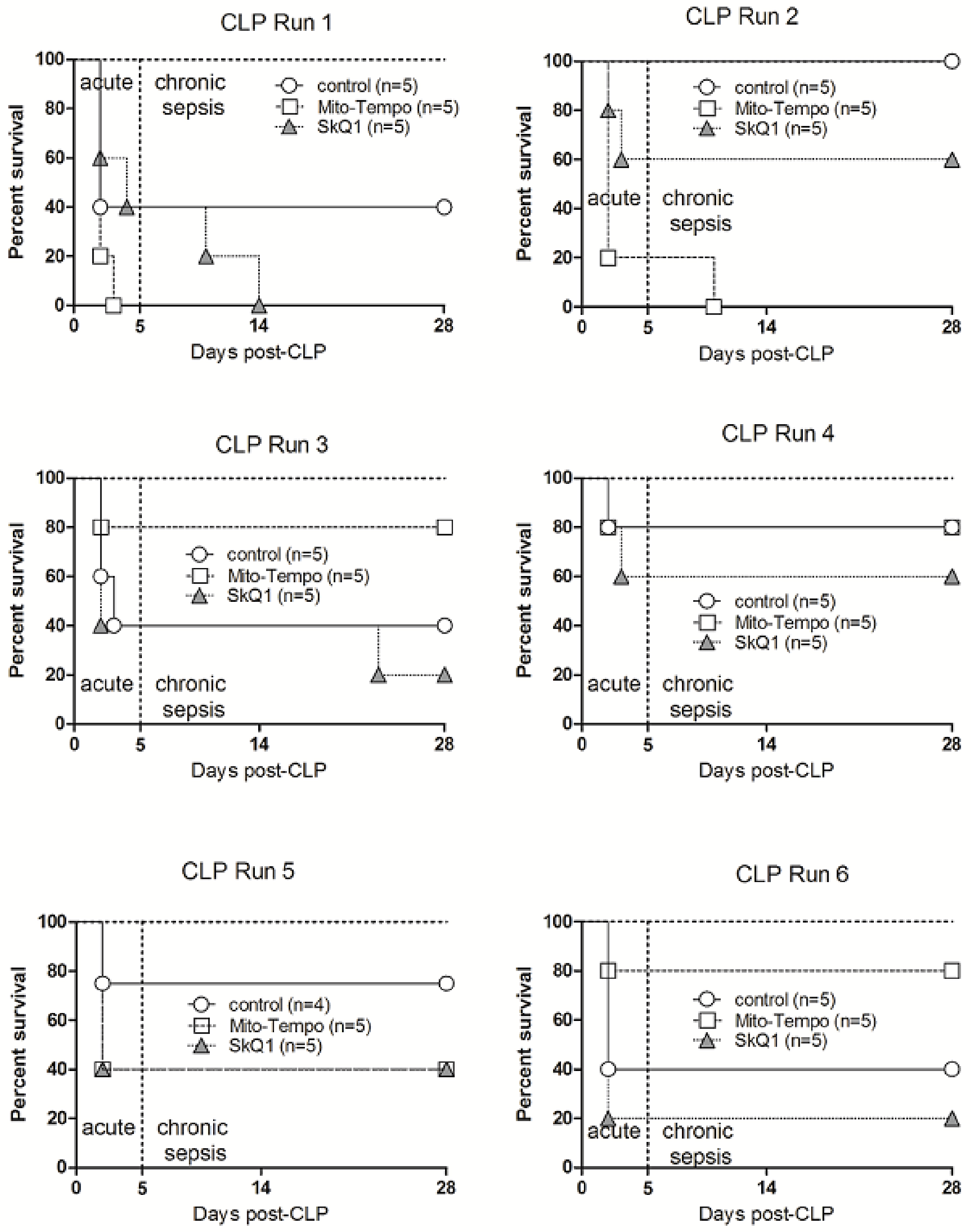

Supplementary Fig. 4. Visualization of outcome for each individual CLP run. CLP was performed in six independent reiterations with 14-15 mice at each repetition (typically 5 mice/each group; the precise n indicated on each panel). Statistical assessment of outcome was performed on the combined data set (Fig. 2).

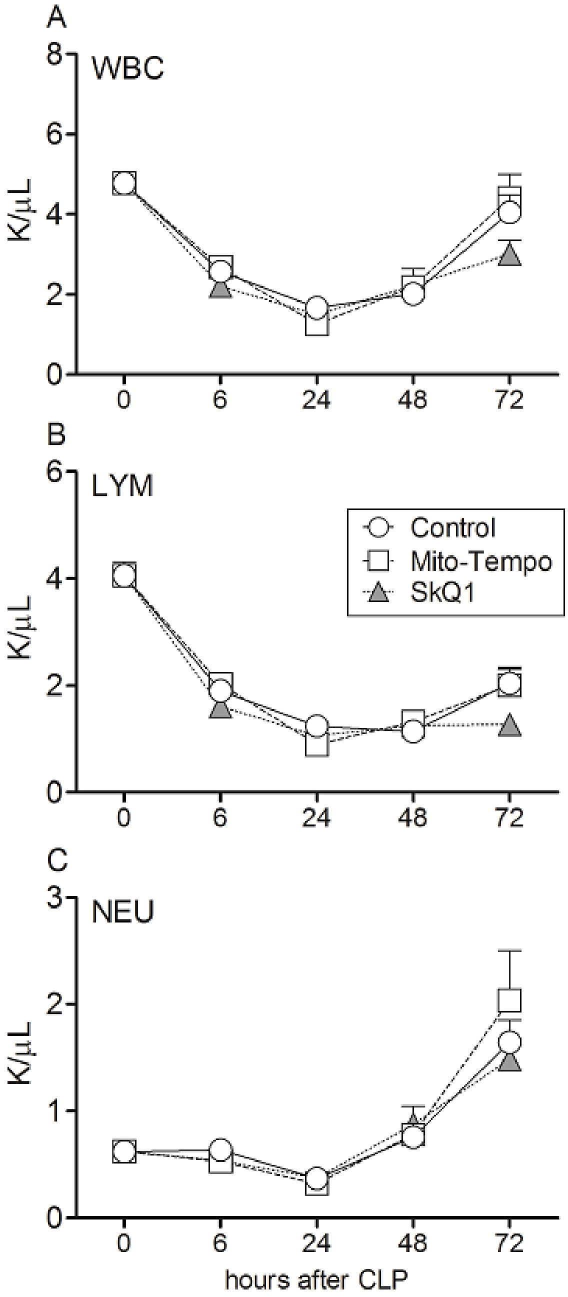

Supplementary Fig. 5. Trajectory of white blood cells (WBC; A), lymphocyte (LYM; B) and neutrophil (NEU; C) counts for SkQ1, MitoTEMPO and placebo mice (control). For **A-C**: at **BL** n=50; at **6h** control n=20, SkQ1 n=25, MitoTEMPO n=23; at **24h** control n=23, SkQ1 n=23, MitoTEMPO n=22; at **48h** control n=18, SkQ1 n=15, MitoTEMPO n=15; at **72h** control n=18, SkQ1 n=14, MitoTEMPO n=15. Data points shown as mean +/- SEM.

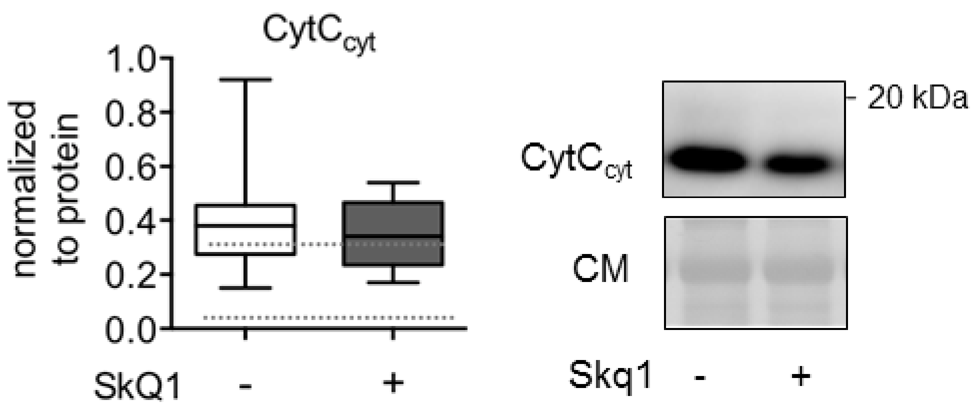

Supplementary Fig. 6. Assessment of Cytochrome C release in the liver of placebo-treated control vs. SkQ1-treated group at 24h post-CLP. CLP mice received total of three SkQ1/placebo injections before sacrifice at 24h. Data is shown as densitometric analysis of the Western blot from cytosolic. Total number of CLP mice loaded on three different gels: SkQ1 n=16; control (placebo) n=12. Data as (min-to-max) box-and-whiskers plots. Dotted lines indicate upper/lower standard deviation calculated based on eight healthy control mice (no CLP, no treatment) that were analyzed together with the CLP mice.CM:.coumassie stained gel.
