# Supplementary material for: Adolescent Trajectories of Aerobic Fitness and Adiposity as Markers of Cardiometabolic Risk in Adulthood
Source: J Obes. 2017 Nov 27;2017:6471938. doi: 10.1155/2017/6471938 (PMC5723934; doi:10.1155/2017/6471938)
Supplement: Supplementary file 3 [file 6471938.f3.docx]

Supplementary Table 1. Descriptive variables for Saskatchewan Growth and Development Study participants who returned for the 2009/10 follow-up study (Responders) and those who did not (Non-Responders). Presented as mean ± standard deviation

|  | Non Responders n= 196^a^ | | | Responders n= 61 | | |
| --- | --- | --- | --- | --- | --- | --- |
| **Adolescent Age (yrs)** | 13.08 | ± | 1.60 | 13.44 | ± | 1.63 |
| **Age of PHV (yrs)** | 13.13 | ± | 1.57 | 13.45 | ± | 1.66 |
| **Height at PHV (cm)** | 156.56 | ± | 10.00 | **160.09** | **±** | **9.78** |
| **Weight at PHV (kg)** | 45.69 | ± | 9.34 | 46.95 | ± | 9.01 |
| **BMI at PHV (kg/m^2^)** | 18.47 | ± | 2.30 | 18.17 | ± | 2.12 |
| **LBM at PHV (kg)** | 39.90 | ± | 5.63 | 40.61 | ± | 5.48 |
| **Percent Body Fat at PHV (%)** | 19.10 | ± | 4.31 | 17.53 | ± | 4.23 |
| **Sum 6 SF at PHV (mm)** | 59.37 | ± | 22.87 | 54.05 | ± | 20.68 |
| **Absolute VO2 at PHV (ml/min)** | 2.29 | ± | 0.69 | 2.42 | ± | 0.77 |
| **Relative VO2 at PHV (ml/kg/min)** | 50.13 | ± | 10.06 | 50.88 | ± | 13.57 |
| **Trunk SF at PHV (mm)** | 17.49 | ± | 9.72 | 15.93 | ± | 7.19 |

Abbreviations found in table. PHV = peak height velocity; BMI = body mass index; LBM = lean body mass; SF = skinfolds. Bolded numbers indicate a significant difference between Responders and Non-Responders.

^a^ not all participants who did not return for the follow up were included in this analysis because they did not have a measure ascertained at the age of peak height velocity.
