# Supplementary material for: Adolescent Trajectories of Aerobic Fitness and Adiposity as Markers of Cardiometabolic Risk in Adulthood
Source: J Obes. 2017 Nov 27;2017:6471938. doi: 10.1155/2017/6471938 (PMC5723934; doi:10.1155/2017/6471938)
Supplement: Supplementary file 4 [file 6471938.f4.docx]

Supplementary Table 2. . Multilevel regression models for growth trajectories of aerobic fitness, adiposity, and obesity status measures for Saskatchewan Growth and Development Study participants who returned in the 2009/10 follow-up study (Responders) and those who did not (Non-Responders).

| **Variable** | **Height** | **Weight** | **AbsVO2** | **Sum6SF** | **TrunkSF** | **BMI** |
| --- | --- | --- | --- | --- | --- | --- |
| ***Fixed Effects*** |  |  |  |  |  |  |
| Constant | 143.18 ± 0.24 | 36.04 ± 0.19 | 1.82 ± 0.02 | 48.03 ± 0.78 | 17.34 ± 7.91 | 17.02 ± 0.07 |
| Biological Age (yrs) | 5.80 ± 0.05 | 3.68 ± 0.05 | 0.20 ± 0.01 | 1.71 ± 0.18 | 1.43 ± 0.14 | 0.5 ± 0.02 |
| Biological Age^2^ (yrs) | NS | NS | NS | NS | NS | NS |
| Sex | NS | NS | -0.20 ± 0.01 | 19.73 ± 1.06 | 11.74 ± 0.86 | NS |
| Responder/Non-Responder | NS | NS | NS | NS | NS | NS |
|  |  |  |  |  |  |  |
| ***Random Effects*** |  |  |  |  |  |  |
| Level 1 |  |  |  |  |  |  |
| Constant (εij) | 21.56 ± 6.53 | 12.17 ± 2.63 | NS | 277.57 ± 80.10 | 157.96± 46.31 | 2.14 ± 0.64 |
| Level 2 |  |  |  |  |  |  |
| Constant (µj) | 24.59 ± 6.62 | 25.36 ± 3.01 | 0.15 ± 0.03 | 228.22 ± 80.77 | 42.17 ± 7.32 | 1.84 ± 0.64 |
| Biological Age (νjXij) | NS | 1.5 ± 0.18 | 0.02 ± 0.001 | NS | NS | NS |
| Constant*Biological Age (µj*νjXij) | NS | 6.48 ± 0.36 | 0.02 ± 0.002 | NS | NS | NS |

All numerical values are reported as significant, *p* < 0.05 (mean > 2*SEE). NS = Not significant and variable removed from the final model

Fixed effect values are Estimated Mean Coefficients ± SEE (Standard Error Estimate) for absolute VO_2_ Height (cm), Weight (kg), (AbsVO_2_, ml/min), sum of six skinfolds (Sum6SF, mm), trunk skinfolds (TruckSF, mm) and body mass index (BMI, kg/m^2^).

Random effects values are Estimated Mean Variance ± SEE.

Biological age is years centered around peak height velocity years of age (yrs). Sex (Male=0, Females =1). Responder ( 0=Non-Responder, 1=Responder).
